# Rat Hepatocytes Protect against Lead–Cadmium-Triggered Apoptosis Based on Autophagy Activation

**DOI:** 10.3390/toxics12040285

**Published:** 2024-04-12

**Authors:** Junshu Xue, Huimao Liu, Tianyi Yin, Xun Zhou, Xu Song, Yuanfeng Zou, Lixia Li, Renyong Jia, Yuping Fu, Xinghong Zhao, Zhongqiong Yin

**Affiliations:** 1 Natural Medicine Research Center, College of Veterinary Medicine, Sichuan Agricultural University, Chengdu 611130, China; 2 Key Laboratory of Animal Disease and Human Health of Sichuan Province, Sichuan Agricultural University, Chengdu 611130, China; xinghong.zhao@sicau.edu.cn (X.Z.);

**Keywords:** lead, cadmium, autophagy, apoptosis, BRL-3A cell, protective

## Abstract

Lead and cadmium are foodborne contaminants that threaten human and animal health. It is well known that lead and cadmium produce hepatotoxicity; however, defense mechanisms against the co-toxic effects of lead and cadmium remain unknown. We investigated the mechanism of autophagy (defense mechanism) against the co-induced toxicity of lead and cadmium in rat hepatocytes (BRL-3A cells). Cultured rat liver BRL-3A cell lines were co-cultured with 10, 20, 40 μM lead and 2.5, 5, 10 μM cadmium alone and in co-culture for 12 h and exposed to 5 mM 3-Methyladenine (3-MA), 10 μM rapamycin (Rapa), and 50 nM Beclin1 siRNA to induce cellular autophagy. Our results show that treatment of BRL-3A cells with lead and cadmium significantly decreased the cell viability, increased intracellular reactive oxygen species levels, decreased mitochondrial membrane potential levels, and induced apoptosis, which are factors leading to liver injury, and cell damage was exacerbated by co-exposure to lead–cadmium. In addition, the results showed that lead and cadmium co-treatment induced autophagy. We further observed that the suppression of autophagy with 3-MA or Beclin1 siRNA promoted lead–cadmium-induced apoptosis, whereas enhancement of autophagy with Rapa suppressed lead–cadmium-induced apoptosis. These results demonstrated that co-treatment with lead and cadmium induces apoptosis in BRL-3A cells. Interestingly, the activation of autophagy provides cells with a self-protective mechanism against induced apoptosis. This study provides insights into the role of autophagy in lead–cadmium-induced apoptosis, which may be beneficial for the treatment of lead–cadmium-induced liver injury.

## 1. Introduction

Lead (Pb) and cadmium (Cd) are frequently detected in food [1,2] and are an ongoing concern worldwide because of their toxicity to humans and animals. People assess Pb or Cd individually; however, Pb and Cd usually coexist in food [3]. Therefore, exploring their common toxicities as well as preventive and curative measures will guide us in addressing health problems associated with Pb and Cd poisoning.

Pb and Cd are concurrent hazardous pollutants in the environment. They mainly accumulate in crops, water sources, and soil and enter the human body through the food chain. When Pb and Cd are ingested via the digestive system or uptake occurs through the skin or respiratory tract, they act as potentially toxic substances that disrupt normal physiological and metabolic body functions [4]. The liver is generally recognized as a major target organ for Pb and Cd. Researchers detected Pb and Cd near the livers of people exposed to mining and smelting and observed that both heavy metals reduced liver function [5]. It has been suggested that the liver contains 33% of the Pb and parts of the Cd that are accumulated when Pb or Cd is inhaled or ingested [6,7]. The results of numerous studies showed that the buildup of Pb and Cd damages the liver, resulting in increased levels of reactive oxygen species (ROS), liver inflammation, hepatic oedema, and necrosis. Researchers reported that rat liver exposure to Pb induces hepatotoxicity by disrupting the mitochondrial respiratory complex [8,9,10]. The results of other studies showed that exposure to Pb and Cd induces cellular autophagy and apoptosis [11,12,13].

Apoptosis is a widespread type of programmed cell death. It Pbs to the renewal of cells during physiological events but also acts as a cellular response to stress [14]. When stimulated by external substances (e.g., heavy metals and viruses) or internal factors produced by the cell itself (e.g., reactive oxygen species and cytotoxins), cells can undergo programmed cell death [15]. Apoptosis is regulated by several genes and pathways. When cells are stimulated, the Bcl-2 protein family triggers a change in the mitochondrial membrane potential (MMP), releasing Cyt c and inducing apoptosis [14]. Exposure of porcine splenocytes to copper increases the expression levels of apoptosis-related Bax, Cyt c, and P53, and induced apoptosis [16]. Many researchers showed that exposure to Pb and Cd Pbs to a significant increase in the expression of the apoptosis-related protein Bax in cells [4,17,18].

Autophagy is a highly conserved eukaryotic recycling process carried out as a lysosomal degradation pathway through the cytoplasm, organelles, proteins, and degradation products transported to the lysosomes [19]. Autophagy plays a crucial role in cell survival. Autophagy provides energy for cell repair and survival when the body is subjected to external stimuli that cause stress, inflammation, and cardiovascular disease [20]. In addition, autophagy maintains homeostasis by eliminating hazardous compounds inside cells and preventing the buildup of damaged organelles. According to previous studies, Beclin-1 and LC3 II proteins, which are involved in autophagy, are upregulated by Pb in chicken testes. Researchers carried out subchronic toxicity experiments and observed that Pb- or Cd-induced elevated levels of autophagy in rat livers [21,22]. Further studies showed that Cd increases liver ROS levels and damages organelles to promote carcinogenesis; however, autophagy effectively reduces ROS and damaged organelles [23]. In another study, cells showed reduced damage from Cd-induced apoptosis after 6 h of Cd treatment due to enhanced autophagy-regulated proteins [24]. However, autophagy is also dysregulated. Several pathological and physiological processes in the body are strongly associated with dysfunctional autophagy, which promotes cancer cell proliferation and spread [25]. Several researchers indicated that autophagy plays a cytotoxic role in apoptosis and exacerbates cell damage [26,27]. In our previous studies, we showed that Pb and Cd induce apoptosis in rat liver in vivo [28]; however, it remains unclear which role autophagy plays in the co-induction of apoptosis in rat hepatocytes by Pb and Cd.

In this study, the normal rat liver cell line BRL-3A was used. We modelled the acute toxicity of Pb and toxicity with Cd in BRL-3A cells in vitro and examined the BRL-3A cell viability, effects of ROS changes, the MMP, apoptosis, and autophagy-related protein expression. After pretreatment with autophagy inhibitors or agonists and Beclin-1 silencing, we also assessed apoptosis-related markers. The objectives of this study were to analyze the function and association of autophagy in Pb- and Cd-induced hepatocyte apoptosis and to provide a rationale for the treatment and prevention of autophagy in heavy metal-induced apoptosis.

## 2. Materials and Methods

### 2.1. Materials

Lead acetate and cadmium chloride (Kelong, Chengdu, China), modified eagle medium (MEM) (C11095500Bt, Gibco, Waltham, MA, USA), Fetal Bovine Serum (FBS) (ST30-3302, PAN SERATECH, Adenbach, Germany), CCK-8 (MA0218, meilunbio, Dalian, China), Monodanoyl cadaverine (MDC) (G0170), DAPI (C0065), Dichlorofluorescin diacetate (DCFH-DA) fluorescence kit (CA1410), JC-1-Mitochondrial Membrane Potential Assay Kit (M8650), ColorMixed Protein Marker (PR1910; PR1930), and phenylmethylsulfonyl fluoride (PMSF) (P0100) were purchased from Solarbio, Beijing China. TRlzol reagent and BCA Protein Assay Kit (Biomed, Beijing, China), ExonScript RT SuperMix with dsDNase (A502-01, Exongen, Chengdu, China), SYBR Green reagents (11201ES, Yeasen, Shanghai China), 3-Methyladenine and rapamycin (HY-19312, HY-10219, MedChemExpress, Monmouth Junction, NJ, USA), Beclin1 si RNA and control si RNA (Tsingke Biotech, Beijing, China), RIPA buffer (P0013B, Beyotime Biotechnology, Shanghai, China), anti-LC3A/B (12741), and anti-p62/SQSTM1 (5114) were purchased from Cell Signaling Technology (Boston, MA, USA). Anti-Beclin1 (11306-1-AP), anti-Bcl2 (26593-1-AP), anti-Bax (50599-2-lg), anti-Cyt c (10993-1-AP), anti-β-actin (20536-1-AP), and horseradish peroxidase (HRP)-coupled goat anti-rabbit lgG (SA00001-2) were purchased from Proteintech (Wuhan, China).

### 2.2. Conditions for Cell Culture and Exposure

**Figure A1:**
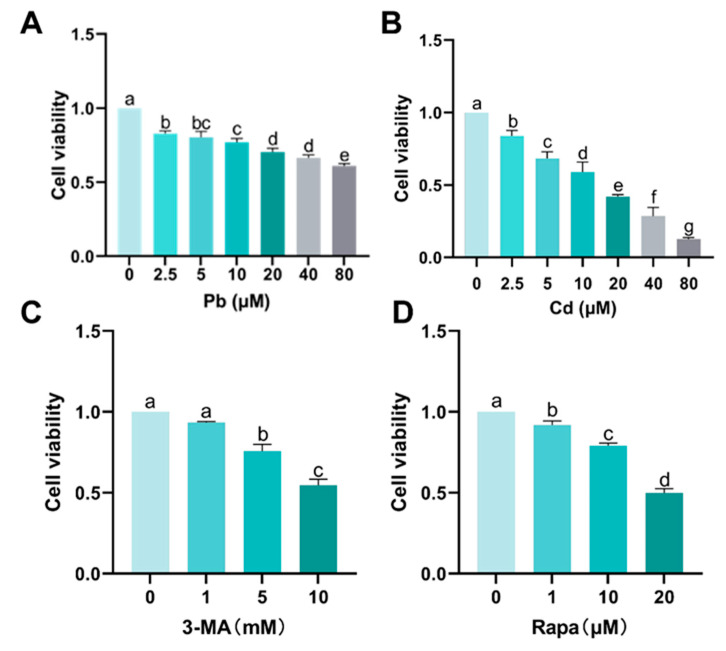
Screening of Pb, Cd, 3-MA, and Rapa dose concentrations. (**A**–**D**) Cell viability by CCK-8 assay. The data are expressed as the mean ± SD (n = 3). Bars with different letters are significantly different (*p* < 0.05).

**Figure A2:**
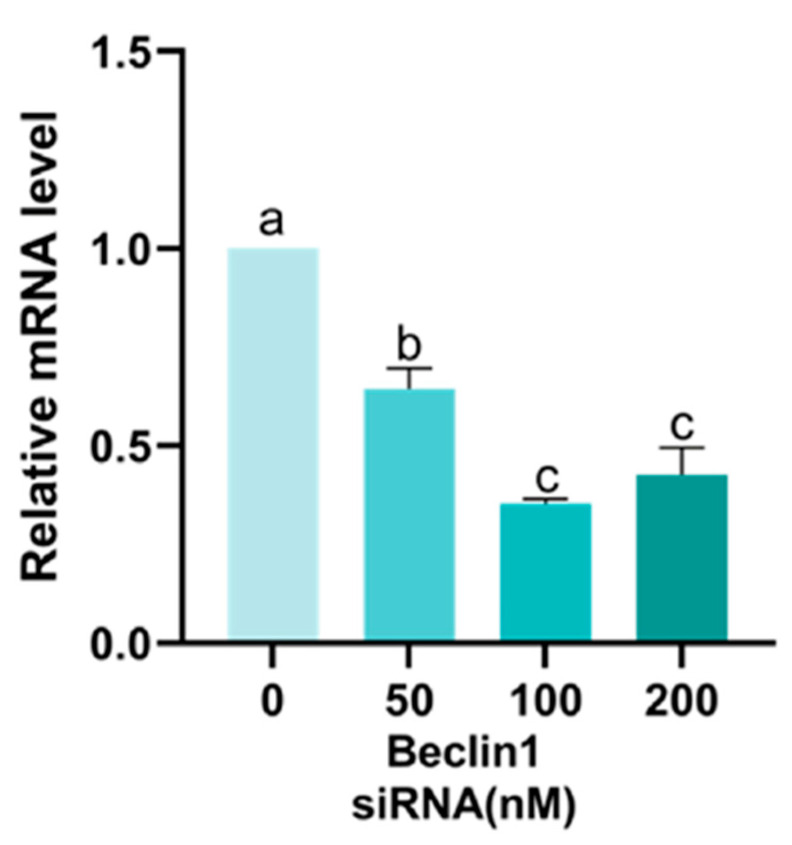
Screening of Beclin1 siRNA dose concentrations. The data are expressed as the mean ± SD (*n* = 3). Bars with different letters are significantly different (*p* < 0.05).

The FBS-supplemented MEM was used to maintain the rat hepatocyte line BRL-3A (C7001) after it was bought from FuHeng Biology (Shanghai, China). We used combined exposure to Pb (AC)_2_ (Pb) and CdCl_2_ (Cd) dissolved in MEM to cause damage to BRL-3A cells. In 96-well plates, 5 × 10^3^ cells suspended in 100 μL of MEM were planted into each well. Cells were cultured for an additional day and then exposed to Pb and Cd (0, 2.5, 5, 10, 20, 40, and 80 μM), 3-MA (1, 5, 10 mM), and Rapa (1, 10, 20 μM) for 12 h; cell viability was assayed with CCK-8 (Figure A1). Cells were treated with Beclin1 siRNA (50, 100, 200 nM) for 4 h, and the expression level of Beclin1 was detected by qRT-PCR (Figure A2). Doses were chosen based on actually cellular viability obtained by testing, as well as references by others [29,30,31,32,33,34,35].

We performed experimental groups: (1) control group (Control), 10 μM Pb group (Pb-L), 20 μM Pb group (Pb-M), 40 μM Pb group (Pb-M), 2.5 μM Cd group (Cd-L), 5 μM Cd group (Cd-M), 10 μM Cd group (Cd-H), 10 μM Pb + 2.5 μM Cd group (Pb + Cd-L), 20 μM Pb + 5 μM Cd group (Pb + Cd-M), 40 μM Pb + 10 μM Cd group (Pb + Cd-H) for 12 h; (2) control group (Control), 40 μM Pb + 10 μM Cd group (Pb + Cd), 5 mM 3-MA group, 10 μM Rapa group, 40 μM Pb + 10 μM Cd + 5 mM 3-MA group (Pb + Cd + 3-MA), 40 μM Pb + 10 μM Cd + 10 μM Rapa group (Pb + Cd + Rapa) for 12 h; (3) control group (Control), 40 μM Pb + 10 μM Cd group (Pb + Cd), 100 nM Beclin1 siRNA group, 40 μM Pb + 10 μM Cd + 100 nM Beclin1 siRNA group (Pb + Cd + Beclin1 siRNA).

### 2.3. Cell Viability Assay

BRL-3A cells were seeded into a 96-well plate and treated with the corresponding amounts of 10, 20, 40 μM Pb and 2.5, 5, 10 μM Cd, with or without 5 mM 3-MA or 10 μM Rapa for 12 h. First, cells in each well were treated for 1 h at 37 °C with a medium containing 100 μL of CCK-8. Then, using a 450 nm wavelength, the OD value was measured at 450 nm wavelength by using a Varioskan Flash (Thermo Scientific, Waltham, MA, USA).

### 2.4. Nuclear Morphological Observations

After the cells were processed on cell crawls, they were treated with 1:1000 concentration of DAPI and immediately visualized by fluorescence microscopy (Nikon, Tokyo Japan) with excitation wavelengths of 340 nm.

### 2.5. AnnexinV-FITC/PI Apoptosis Analysis

After collecting 5 × 10^5^ cells, subsequent steps are the same as for other researchers [26].

### 2.6. Detection of ROS Level

Treated cells stained for 40 min depending on the instructions of the DCFH-DA fluorescence kit and immediately observed under a fluorescent microscope (Nikon, Japan) with excitation wavelengths of 488 and 525 nm.

### 2.7. Mitochondrial Membrane Potential (MMP) Determination

The cells were treated, then treated according to JC-1-Mitochondrial Membrane Potential Assay Kit and immediately viewed through a fluorescent microscope (Nikon, Japan) with an excitation wavelength of 515 nm and 585 nm.

### 2.8. MDC-Stained Observation of Autophagy Vesicles

Cells were co-treated with Pb and Cd for 12 h. They were stained for 30 min in the dark with 10 μL MDC Stain. Cells were washed and immediately visualized under fluorescence microscopy (Nikon, Japan) of 340 nm.

### 2.9. LC3 Immunofluorescence Aggregation Site Study

The specific method was the same as that used in [36]. Cells were incubated with anti-LC3A/B (1:50) overnight at 4 °C, followed by a secondary antibody (1:1000) for 1 h at room temperature. Lastly, fluorescent microscope pictures were taken (Zeiss, Oberkochen, Germany) of 340 nm and 650 nm.

### 2.10. Transcriptomics

The cells were treated at a concentration of 40 + 10 μM Pb + Cd. Cells without Pb + Cd treatment were used as untreated controls. The entire transcriptome analysis step in this study was performed by Novo to Source Biotechnology (Beijing, China). Briefly, the illumina platform was used to filter and collate the sequencing data, and edgeR was applied to analyze the differentially expressed genes. Magic Novogene was used for DEG volcano mapping and enrichment in the Go database. Three independent biological replicates were performed for each treatment.

### 2.11. Real-Time Quantitative PCR Fluorescence Analysis

Using TRlzol reagent, total RNA was isolated from BRL-3A cells. Then, cDNAs were synthesized using the ExonScript RT SuperMix with dsDNase. The CFX ligated real-time system and SYBR Green RT-PCR reagents were used to conduct the experiment. Table 1 contains a list of primers. RT-PCR cycles were 3 min at 95 °C followed by 39 cycles of 10 s at 95 °C and 30 s at 54 °C. Data were analyzed by the 2^−ΔΔCt^ method and normalized to BRL-3A cells. Data were normalized to BRL-3A cells β-actin.

### 2.12. Western Blot Analysis

Refer to other researchers’ experimental steps [36]. The membranes were exposed to diluted primary antibodies against the target proteins: anti-Beclin1 (1:2000), anti-LC3A/B (1:1000), anti-SQSTM1/p62 (1:1000), anti-Bcl-2 (1:2000), anti-Bax (1:2000), anti-Cyt c (1:1000) at 4 °C overnight, together with the corresponding HRP-coupled secondary antibodies (1:5000) for 1 h. Signals were imaged using a biorad ChemiDoc MP imager (Bio-Rad, Hercules, CA, USA). Finally, the grey scale values of the corresponding proteins were analyzed by Image J2.

### 2.13. Statistical Analysis

All experiments were performed three times and statistical analysis was performed using SPSS 23.0 and GraphPad Prism 9.0. All data are expressed as mean ± standard deviation (SD). One-way analysis of variance (ANOVA) and least significant difference (LSD) post hoc tests were used to examine the data. Significant results were defined as having a *p*-value of less than 0.05 (*p* < 0.05).

## 3. Results

### 3.1. Cytotoxicity of Pb and Cd in BRL-3A Cells

**Figure 1:**
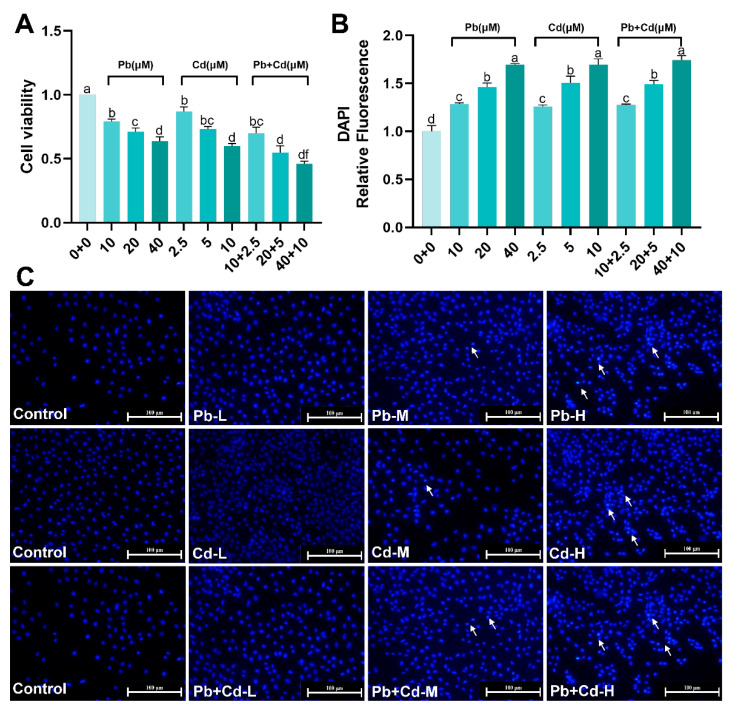
Effects of Pb and Cd on cytotoxicity. (**A**) Cell viability. (**B**) Quantitative analysis of DAPI fluorescence intensity in the BRL-3A cells. (**C**) Apoptosis was measured by using DAPI staining in the BRL-3A cells (scale bar = 100 μm). Blue-fluorescent dots indicate apoptotic vesicles. Arrows indicate that the nucleus undergoes cleavage and shrinkage. The data are expressed as the mean ± SD (*n* = 3). Bars with different letters are significantly different (*p* < 0.05).

Based on a set gradient of Pb and Cd concentrations, we examined the effect of 12 h Pb–Cd exposure on cell viability (Figure 1A). The results showed that the cell viability of all Pb- and Cd-treated groups significantly decreased compared with that of the control group (*p* < 0.01) and a dose–effect relationship was observed. Furthermore, cell activity was significantly lower in the 20 + 5 μM Pb + Cd group compared to in the 20 μM Pb or 5 μM Cd groups (*p* < 0.01). DAPI staining indicated that the control group had a normal nucleus, whereas the nuclei of the Pb- and Cd-treated groups exhibited significant changes. These changes included condensation in the nucleus and crescentic chromatin in several nuclei (Figure 1C). Figure 1B showed that the fluorescence intensity of all Pb and Cd groups was significantly enhanced compared to that of the control group (*p* < 0.05). There was no significant difference in the fluorescence intensity of the Pb + Cd group at the corresponding dose compared to that of the Pb or Cd groups (*p* > 0.05). These results indicate that Pb and Cd caused cytotoxicity and cellular damage, and combined exposure to Pb + Cd can Pb to worsening cell damage.

### 3.2. Pb and Cd Cause ROS and MMP Changes in BRL-3A Cells

**Figure 2:**
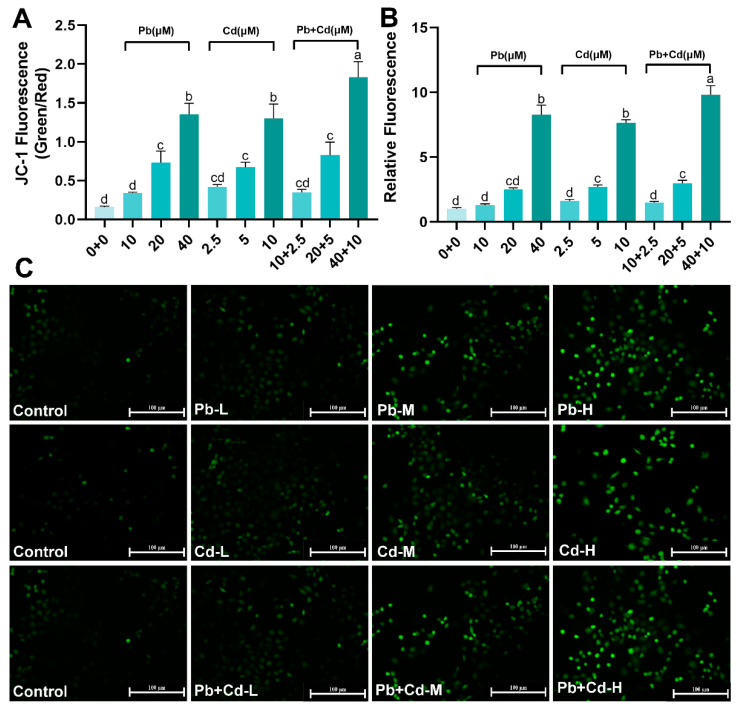
Effects of Pb and Cd on ROS and MMP level in BRL-3A cells. (**A**) Fluorescence quantification of MMP in BRL-3A cells after exposure to Pb and Cd. Decrease in MMP indicated by the shift from red to green fluorescence of JC-1. (**B**) Fluorescence quantification of ROS in BRL-3A cells after exposure to Pb and Cd. (**C**) The ROS expression level of the BRL-3A cells after Pb and Cd exposure (scale bar = 100 μm). The green fluorescence revealed the level of ROS. The data are expressed as the mean ± SD (*n* = 3). Bars with different letters are significantly different (*p* < 0.05).

Because ROS formation is a consequence of the body’s reaction to external stimuli, an increase in ROS levels can lead to changes in the MMP. The level of intracellular ROS accumulation was detected using DCFH-DA fluorescent probes specific to reactive oxygen species. Figure 2B,C show that the green fluorescence of BRL-3A cells in the 40 μM Pb and 5, 10 μM Cd and 20 + 5, 40 + 10 μM Pb + Cd groups significantly increased compared with the control group (*p* < 0.05). Compared with the 40 µM Pb group and the 10 µM Cd group, the green fluorescence of the 40 + 10 µM Pb + Cd group was clearly enhanced. Exposure to Pb and Cd increased cellular ROS levels. In addition, we evaluated the intracellular MMP-ΔΨm using the MMP-specific JC-1 fluorescent probe. The results of staining with the JC-1 probe revealed that an increase in the Pb–Cd dose was accompanied by an increase in the intensity of green fluorescence and a decrease in the intensity of red fluorescence and Pb and Cd exposure clearly reduced MMP levels in BRL-3A cells compared with those in the control group (Figure S1). Meanwhile, the ratio of green and red fluorescence intensity significantly increased in the 40 + 10 µM Pb + Cd group compared to that in the 40 µM Pb and 10 µM Cd groups (Figure 2A). These results indicate that exposure of BRL-3A cells to Pb and Cd can lead to oxidative stress and mitochondrial damage.

### 3.3. Pb and Cd Induce Apoptosis in BRL-3A Cells

**Figure 3:**
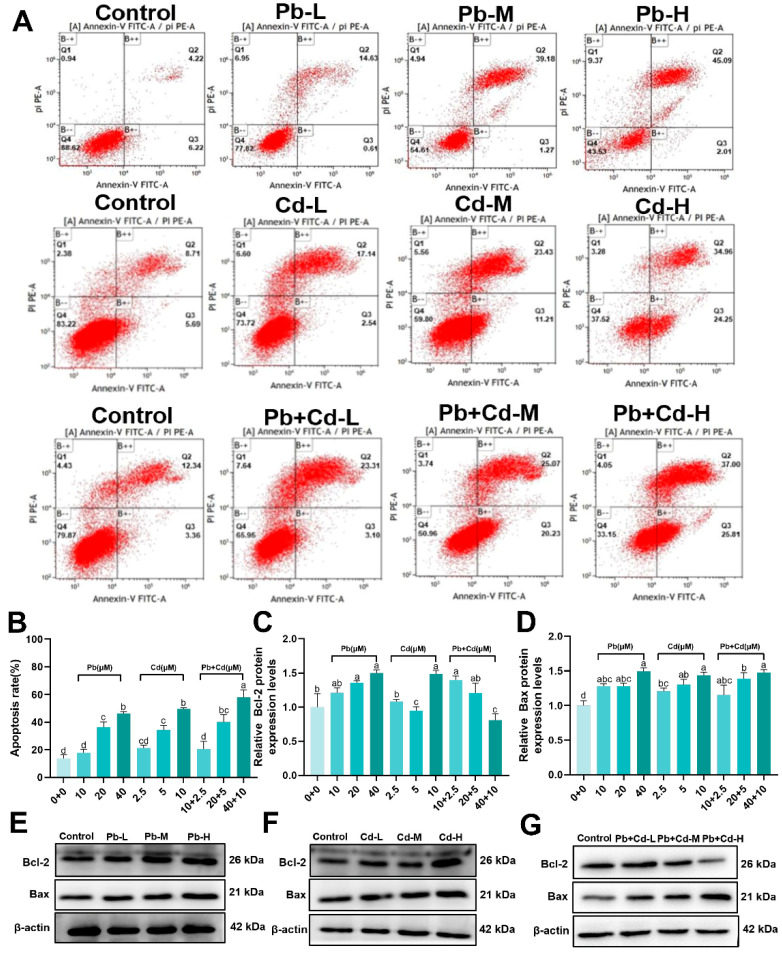
Effects of Pb and Cd on apoptosis in BRL-3A cells. (**A**,**B**) Annexin V-FITC flow analysis and the percentage of apoptotic cells per total population was detected. (**C**–**G**) The protein levels of apoptosis-related genes (Bcl-2, Bax). The data are expressed as the mean ± SD (*n* = 3). Bars with different letters are significantly different (*p* < 0.05).

To further explore the effect of Pb and Cd exposure on apoptosis, flow cytometry and Western blotting were performed. As shown in Figure 3A, compared with the control group, the apoptosis rates of early and late apoptotic cells of membrane-associated proteins in the 20, 40 μM Pb and 10 μM Cd and 20 + 5, 40 + 10 μM Pb + Cd treatment groups were significantly higher (*p* < 0.05). The rate of apoptosis significantly increased in the group exposed to 40 + 10 µM Pb + Cd compared to in the groups exposed to 40 µM Pb and 10 µM Cd. (*p* < 0.05; Figure 3A,B.)

As shown in Figure 3C–G, the study found that exposure to Pb and Cd resulted in increased relative expression of Bax protein in cells exposed to 10, 20, 40 μM Pb and 2.5, 5, 10 μM Cd alone and in combination compared to in the control group (*p* < 0.05). Additionally, the relative expression of the Bcl-2 protein was significantly lower (*p* < 0.05) in cells exposed to 5 μM Cd and 40 + 10 μM Pb + Cd groups. However, the relative expression of Bax protein did not significantly change with the corresponding doses of combined Pb and Cd exposure compared to single Pb and Cd exposure (*p* > 0.05). The study concludes that Pb and Cd exposure induces apoptosis in BRL-3A cells.

### 3.4. Pb and Cd Trigger Autophagy in BRL-3A Cells

**Figure 4:**
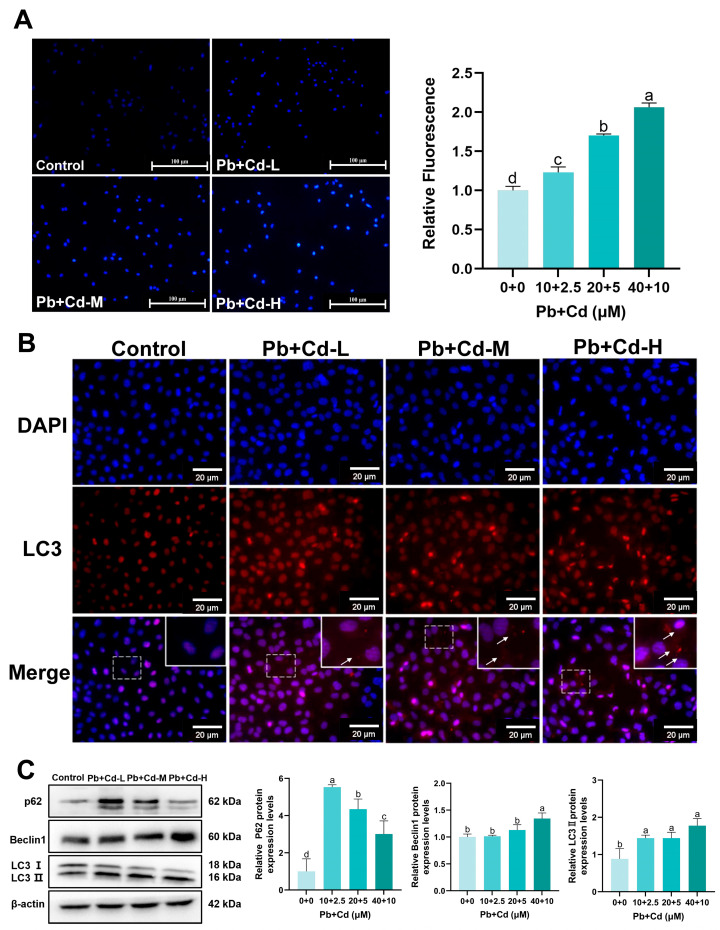
Effects of Pb and Cd on autophagy in BRL-3A cells. (**A**) Autophagy was measured by using MDC staining in the BRL-3A cells (scale bar = 100 μm). (**B**) LC3 immunofluorescence labeling in BRL-3A cells (scale bar = 20 μm). The arrows point to the red fluorescent aggregates of LC3. Dashed box for image enlargement. (**C**) The protein levels of autophagy-related genes (p62, Beclin1, LC3Ⅱ). The data are expressed as the mean ± SD (*n* = 3). Bars with different letters are significantly different (*p* < 0.05).

MDC staining was used to observe autophagosomes in BRL-3A cells. A large number of blue-fluorescent dot-like structures appeared in Pb–Cd-co-exposed cells compared with in the control group (Figure 4A). Figure 4B showed the LC3 immunofluorescence results. The number of LC3 dots significantly increased in the 20 and 40 μM Pb + Cd groups compared with in the control group. LC3 cumulative points showed a dose–effect relationship. To further determine the effects of combined Pb and Cd exposure on autophagy in BRL-3A cells, we examined the levels of autophagy-related proteins by Western blotting of BRL-3A cells. Figure 4C revealed that the protein levels of LC3II and p62 were significantly increased in the Pb-Cd group and the protein levels of Beclin1 were significantly increased in the 40 μM Pb + Cd group compared with those in the control group (*p* < 0.05). Our results show that Pb and Cd Pb to autophagy in BRL-3A cells.

### 3.5. GO Enrichment Analysis and Transcriptomic Validation of DEGs

**Figure 5:**
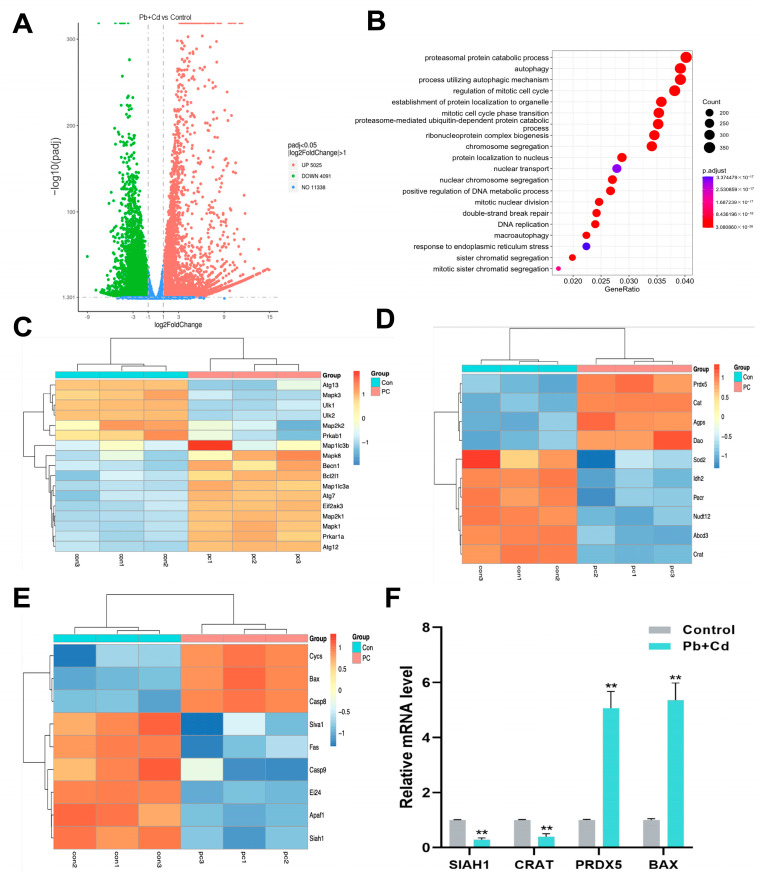
Transcriptome analysis of BRL-3A cells after Pb and Cd co-staining. (**A**) Volcano 20 plot of DEGs. (**B**) Scatterplot of Go enrichment analysis. (**C**–**E**) Heatmap of genes associated with the autophagy–peroxisome–P53 pathway. (**F**) Correlation of expression level analyzed by qRT-PCR with data obtained using RNA-Seq platform. The data are expressed as the mean ± SD (*n* = 3). ** *p* < 0.01.

We further validated the results of preliminary experiments using transcriptome analysis. After 12 h of combined Pb and Cd treatment, significant transcriptional alterations were observed in BRL-3A cells (Figure 5). In total, 9116 differentially expressed genes (DEGs), among which 5025 were upregulated and 4091 were downregulated, were observed in BRL-3A cells co-treated with Pb and Cd compared with those in the control group (Figure 5A) {(log2 fold change) > 1, *p* < 0.05}. GO enrichment analysis revealed that autophagy was significantly enriched among the top 20 pathways (Figure 5B). According to the heatmap analysis of autophagy (Figure 5C), peroxisome (Figure 5D), and P53 (Figure 5E) signaling pathways, the autophagy-related gene Beclin1 and apoptosis-related gene Bax were significantly upregulated compared with the control group, and peroxisomes were closely related to the ROS level. The results showed combined exposure to Pb- and Cd-induced oxidative stress, apoptosis, and autophagy in BRL-3A cells. These results are consistent with those of our previous experiments.

Quantitative real-time polymerase chain reaction (qRT-PCR) was used to assess the mRNA levels of several DEGs with |log2 (fold-change)| > 2 to verify the reliability of our transcriptome data. Figure 4F shows that, compared with untreated cells, PRDX5 and Bax mRNA levels in Pb–Cd-treated cells were increased and CRAT and SIAH1 mRNA levels were notably downregulated (*p* < 0.05). RNA sequencing data agreed with the patterns of the values of each gene. This confirms the validity of our transcriptomic results.

### 3.6. Autophagy Plays a Protective Role in BRL-3A Cells Exposed to Pb and Cd

**Figure 6:**
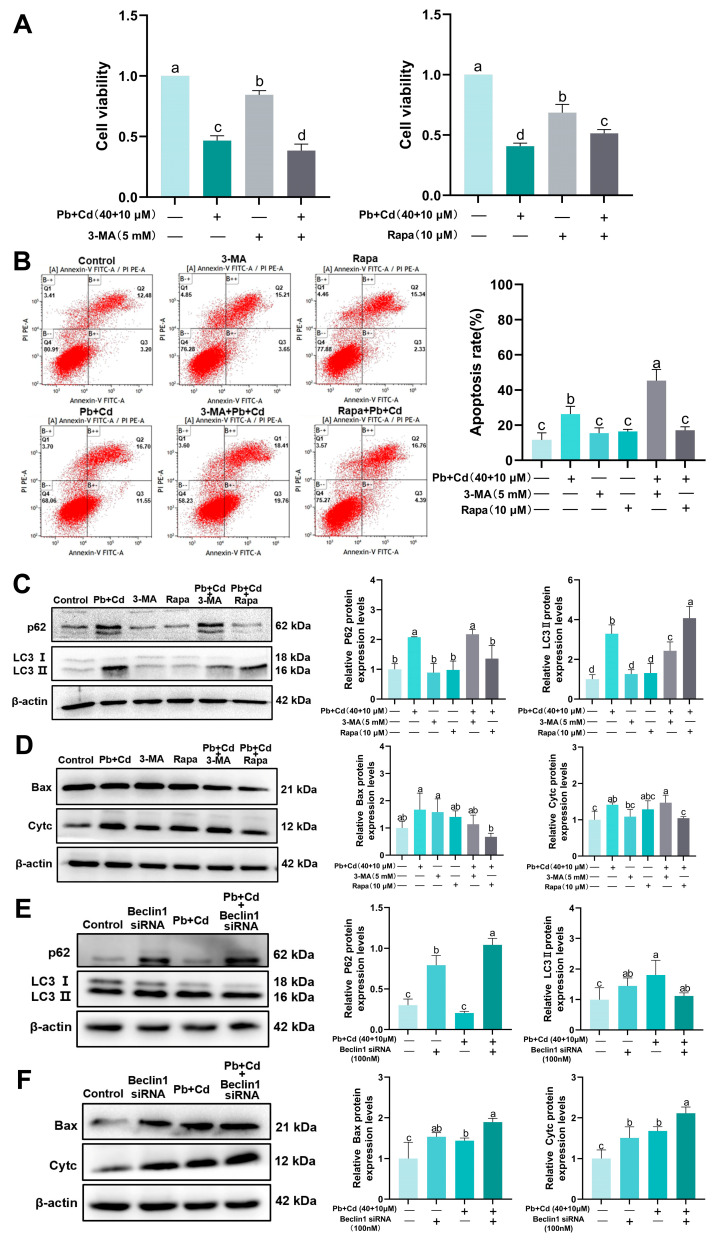
Effects of 3-MA, Rapa, and Beclin1 siRNA transfection in BRL-3A cells. (**A**) Cell viability by CCK-8 assay. (**B**) The fraction of apoptotic cells per total population was determined using Annexin V-FITC flow analysis. (**C**,**E**) The protein levels of autophagy-related genes (p62, LC3Ⅱ). (**D**,**F**) The protein levels of apoptosis-related genes (Bax, Cyt c). The data are expressed as the mean ± SD (*n* = 3). Bars with different letters are significantly different (*p* < 0.05).

Based on the results of previous experiments, we further explored the relationship between cellular autophagy and apoptosis under Pb–Cd co-exposure conditions using an autophagy intervention assay. Figure 6A,B show that the cell viability and apoptosis rate were inhibited by 3-MA compared with the Pb–Cd co-treatment groups and that Rapa mitigated the toxicity of the Pb–Cd combination. The Pb + Cd + Rapa group showed a significant decrease in p62, Bax, and Cyt c protein levels and a significant increase in LC3II protein levels compared to those of the Pb-Cd co-treatment group (*p* < 0.05; Figure 6C,D). These results suggest that promoting autophagy may inhibit the combined induction of apoptosis in BRL-3A cells by Pb and Cd.

We further discovered that downregulation of Beclin1 by Beclin1 siRNA transfection effectively increased the p62 protein levels induced by Pb–Cd co-exposure (*p* < 0.05; Figure 6E), whereas apoptosis was significantly increased in the Pb + Cd + Beclin1 siRNA group compared with that in the Pb and Cd groups (*p* < 0.05; Figure 6F). These results indicate that the activation of autophagy provides a protection mechanism against the combined induction of apoptosis by Pb and Cd in BRL-3A cells.

## 4. Discussion

Pb and Cd, which are heavy metals widely present in the food chain, may harm the liver when encountered over a long period [37,38]. Most researchers have focused on hepatocyte damage caused by Pb or Cd alone; however, few investigations have been conducted on hepatocyte damage generated by Pb–Cd co-exposure. We observed that Pb and Cd decrease the viability of BRL-3A cells and cause severe morphological alterations in the cell nucleus. Co-exposure to Pb and Cd resulted in increased BRL cells damage compared to exposure to either Pb or Cd alone. Our results show that Pb and Cd are highly cytotoxic in BRL-3A cells. Exposure to Pb and Cd induces oxidative stress, mitochondrial swelling, and disturbances of the energy metabolism in the liver [9,39,40]. Other researchers demonstrated that Pb and Cd can incite hepatic oxidative stress and liver dysfunction in rats in vivo [22]. Accumulation of Pb and Cd in BRL-3A cells causes redox disturbances and increased ROS levels. Furthermore, it has been suggested that excessive ROS may Pb to mitochondrial dysfunction [41]. Based on the results of this study, exposure to Pb and Cd resulted in mitochondrial damage in BRL-3A cells, as evidenced by a decline in the mitochondrial membrane potential, which is consistent with the results of “Cd-alone” staining for BEAS-2B cytotoxicity [42]. In combination with the literature, our results suggest that Pb and Cd toxicity in BRL-3A cells triggers oxidative stress and mitochondrial damage. Among them, the BRL-3A cells exposed to both Pb and Cd showed higher levels of ROS and lower levels of the MMP compared to those of cells exposed to only 40 μM Pb or 10 μM Cd. This indicates that combined exposure to these heavy metals increased the cellular damage caused by single exposure to Pb and Cd. Excessive ROS generation affects mitochondrial homeostasis and plays a role in apoptosis regulation [43]. When cells are stimulated, Pbing to an increase in intracellular ROS, further alterations in the mitochondrial membrane potential are induced, Pbing to apoptosis. It has been reported that heavy metals, such as copper and arsenic, induce elevated levels of cellular ROS, which in turn induce apoptosis [44,45]. The Bcl-2 protein family plays a central role in mitochondrial membrane permeability and Cyt c release during the apoptotic cascade response [46]. Bax is a pro-apoptotic protein; when cells are encapsulated, it is overexpressed to promote apoptosis. According to our study, exposure to Pb and Cd causes a concentration-dependent induction of apoptosis in BRL-3A cells. It is found that co-exposure to Pb and Cd resulted in exacerbated apoptosis when compared to single exposure to 40 μM Pb and 10 μM Cd, indicating a toxic synergistic effect [47]. Our data show that exposure of BRL-3A cells to Pb and Cd results in elevated Bax protein expression, while 10 + 40 μM Pb and Cd results in decreased Bcl-2 protein expression. Taken together, we found that Pb and Cd exposure induced apoptosis and led to cellular damage and that this change was exacerbated by combined Pb and Cd exposure. We analyzed changes in the transcript levels of cellular genes under combined exposure conditions of 10 + 40 μM Pb and Cd by transcriptomics. The P53 signaling pathway also regulates apoptosis-related genes [48]. According to the transcriptome results, combined exposure to Pb and Cd led to significant enrichment of the P53 signaling pathway in BRL-3A cells, and the apoptosis-related gene Bax was significantly upregulated, which verified the above-mentioned results. These results are consistent with those of our previous studies of Pb and Cd-exposed rats and are encouraging [28].

Autophagy is a crucial intracellular breakdown pathway that maintains the homeostatic balance in an organism [49]. However, it remains unclear whether autophagy is activated or inhibited during heavy metal poisoning [50,51]. In mouse kidneys and rat osteoblasts, Pb and Cd enhance the activation of autophagy [52,53]. Other studies showed that Cd and Pb inhibit autophagosomal degradation, Pbing to liver damage. The primary feature of autophagy is the presence of autophagic lysosomes. Autophagic lysosomes are characterized by the presence of key membrane proteins such as LC3 (LC3II and LC3I). Autophagy generally occurs during the conversion of LC3I to LC3II [53]. Autophagosome production is indicated by the ratio of LC3II. In the present study, we examined Pb and Cd in BRL-3A cells and observed that Pb and Cd significantly increased the number of autophagosomes and LC3 accumulation sites and upregulated two specific autophagy markers, which are Beclin1 and LC3II proteins. Interestingly, we observed that protein p62 was upregulated, which is consistent with the results of other researchers [22] who reported that p62 expression was elevated and autophagosome degradation was impaired by the exposure of rats to Pb and Cd for 12 weeks. However, p62 protein levels showed a decreasing trend during normal autophagy, which inspired us to explore the mechanism of autophagosome degradation. In addition, our transcriptomic data indicated significant enrichment of the autophagy signaling pathway, with increased expression of Beclin-1 in Pb- and Cd-treated groups. This further validates our findings. Overall, these results suggest that Pb and Cd trigger autophagy.

The regulatory mechanism of autophagy is complex and its function in apoptosis is not fully understood. Autophagy protects cells from apoptosis and other stimuli by destroying damaged organelles or increasing the autophagic flux inside the cell [54,55]. Notably, when activated under other circumstances, autophagy can also Pb to cytotoxicity [56]. In a subsequent study, we investigated 3-MA and the autophagy agonist Rapa to further elucidate the modulatory effects of autophagy on Pb–Cd-co-induced apoptosis in rat hepatocytes. According to our results, inhibition of autophagy increases Pb- and Cd-induced apoptosis in BRL-3A cells. We further validated these results by silencing the autophagy-related gene Beclin1. As predicted, the activation of autophagy attenuated the apoptosis of BRL-3A cells induced by Pb and Cd. Researchers agree that autophagy has a protective effect on Cd-induced germ cell apoptosis in Sertoli cells. [57]. In addition, it has been reported that the inhibition of autophagy exacerbated apoptosis caused by Cd exposure in duck kidney cells [36]. In conclusion, the role of autophagy in regulating apoptosis is related to the toxicity mechanism of Pb–Cd-co-exposure-induced apoptosis in BRL-3A cells. The results of this study provide a theoretical basis for the treatment of Pb–Cd-co-exposure-induced hepatocyte apoptosis.

## 5. Conclusions

Co-exposure to Pb and Cd can cause oxidative stress, mitochondrial damage, apoptosis, and autophagy in BRL-3A cells, and the activation of autophagy provides a protective mechanism in Pb–Cd-induced apoptosis. Our data suggest that autophagy protects BRL-3A cells from apoptosis upon exposure to Pb and Cd.

## Figures and Tables

**Table 1:** The primers used for qRT-PCR.

Gene Name	Primer Sequence (5′ to 3′)
Beclin1	Forward: AGGAGTTGCCGTTGTACTGTTCTG Reverse: TGCCTCCAGTGTCTTCAATCTTGC
BAX	Forward: CCAGGACGCATCCACCAAGAAGC Reverse: TGCCACACGGAAGAAGACCTCTCG
CRAT	Forward: CAAGCAGGACTTCATGGATCTACAG Reverse: GGCAGCGTCTCGTTGTCAATC
PRDX5	Forward: CCACCAGGCAGAAGGCAAGG Reverse: CGATTCCCAAAGAGAGACACCAAAG
SIAH1	Forward: CAAAGTGTCCACCATCCCAGAG Reverse: GGTGGCAATACATAGTCAAAGCAG
β-actin	Forward: CTAAGGCCAACCGTGAAAAG Reverse: AACACAGCCTGGATGGCTAC

## Data Availability

Some or all data that support the findings of this study are available from the corresponding author upon reasonable request.

## Supplementary Materials

The following supporting information can be downloaded at: https://www.mdpi.com/article/10.3390/toxics12040285/s1, Figure S1: Effects of Pb and Cd on MMP level in BRL-3A cells.

## Appendix A

### Appendix A.1. Screening for Pb, Cd, 3-MA, and Rapa Concentrations

According to the set Pb and Cd concentration gradients, we examined the effect of Pb or Cd exposure for 12 h on cell viability (Figure A1A,B). Cell viability was significantly (*p* < 0.05) reduced in all Pb and Cd individually treated groups compared with in the control group. Based on the experimental results and references to the related literature [35,55], the dose concentration with research significance at ≥50% cell activity was selected. Pb at 10, 20, 40 μM and Cd at 2.5, 5, 10 μM were used as subsequent experimental doses.

We performed cell activity assays for 3-MA and Rapa. According to the experimental results (Figure A1C,D), there was no significant change in cell activity in the 3-MA group of 1 mM compared with in the control group (*p* > 0.05), and the cell activity was significantly reduced in the 3-MA groups of 5 mM and 10 mM (*p* < 0.01). After the cells were treated with Rapa, the cell activity was significantly reduced (*p* < 0.05). Meanwhile, 10 mM 3-MA and 20 μM Rapa had obvious cytotoxicity. Based on the references [56,57] as well as the experimental results, 5 mM of 3-MA and 10 μM of Rapa were finally selected for the subsequent experiments.

### Appendix A.2. Screening of Beclin1 siRNA Concentration

The efficiency of silencing the Beclin1 gene by 50, 100, 200 nM of Beclin1 siRNA was examined by qRT-PCR (Figure A2). Compared with that of the control group, the mRNA expression level of Beclin1 was significantly reduced after silencing by 50, 100, 200 nM Beclin1 siRNA (*p* < 0.01), and the highest efficiency of silencing Beclin1 was achieved by 100 nM Beclin1 siRNA. Based on the experimental results, we chose 100 nM Beclin1 siRNA for subsequent experiments.
