# Supplementary material for: Rat Hepatocytes Protect against Lead–Cadmium-Triggered Apoptosis Based on Autophagy Activation
Source: Toxics. 2024 Apr 12;12(4):285. doi: 10.3390/toxics12040285 (PMC11055059; doi:10.3390/toxics12040285)
Supplement: Supplementary file 1 [file toxics-12-00285-s001.zip › toxics-2937586-supplementary.pdf]

Rat Hepatocytes Protect against Lead–Cadmium-Triggered Apoptosis Based on Autophagy Activation

Junshu Xue[1†], Huimao Liu[1†], Tianyi Yin[1†], Xun Zhou[1], Xu Song[1], Yuanfeng Zou[1], Lixia Li[1], Renyong Jia[2], Yuping Fu[1], Xinghong Zhao[1], Zhongqiong Yin[1*]

[1] Natural Medicine Research Center, College of Veterinary Medicine, Sichuan Agricultural University, Chengdu, 611130, China

[2] Key Laboratory of Animal Disease and Human Health of Sichuan Province, Sichuan Agricultural University, Chengdu, 611130, China

[*] Corresponding author. E-mail: yinzhongq@163.com

[†] These authors contributed equally to this work

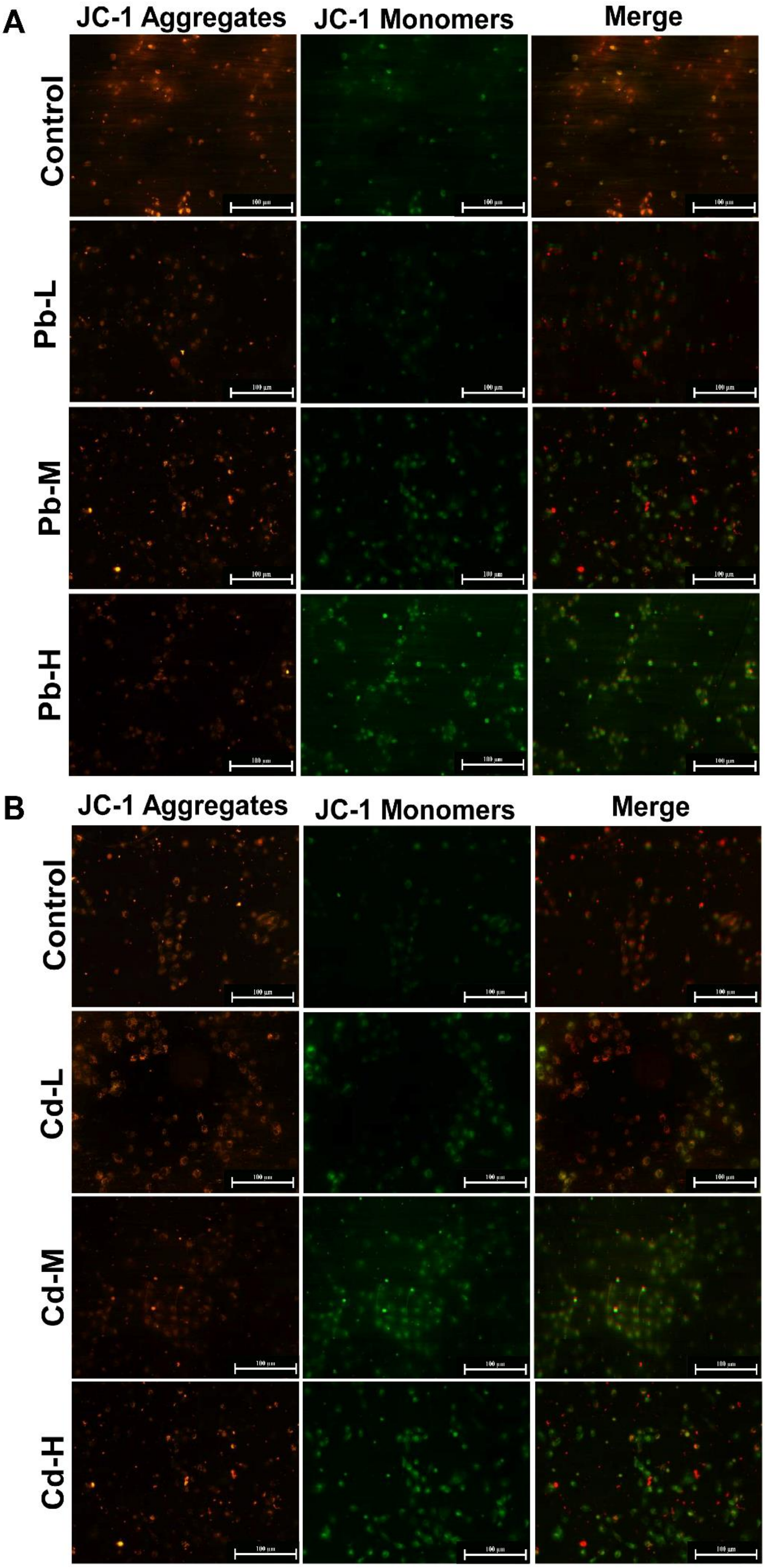
A
JC-1 Aggregates
JC-1 Monomers
Merge
Control
Pb-L
Pb-M
Pb-H
B
JC-1 Aggregates
JC-1 Monomers
Merge
Control
Cd-L
Cd-M
Cd-H
100 μm

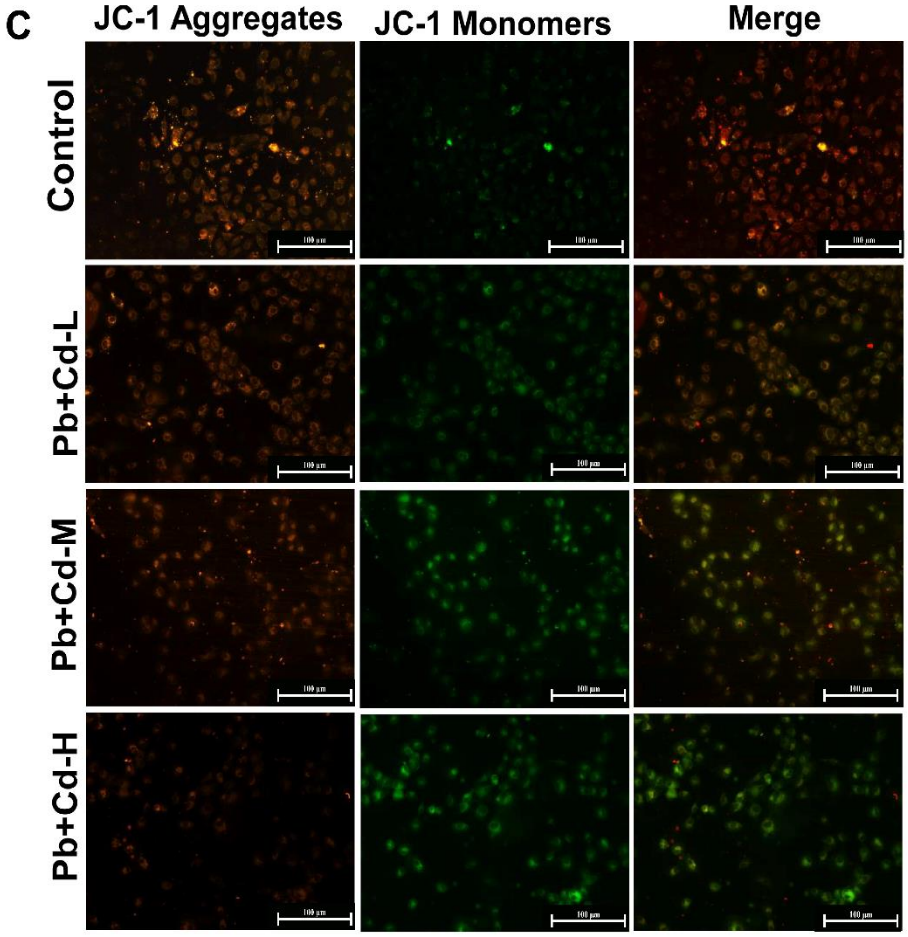

Figure S1. Effects of Pb and Cd on MMP level in BRL-3A cells. (**A–C**) The MMP expression level of the BRL-3A cells after Pb and Cd exposure (scale bar = 100 μm). Decrease in MMP indicated by the shift from red to green fluorescence of JC-1.
